# The impact of simultaneous inoculation with *Torulaspora delbrueckii* and *Hanseniaspora uvarum* combined with *Saccharomyces cerevisiae* on chemical and sensory quality of Sauvignon blanc wines

**DOI:** 10.3389/fmicb.2024.1413650

**Published:** 2024-07-24

**Authors:** Linbo Li, Chenyang Yuan, Lei Zhang, Ruichao Chu, Qingquan Yu, Jian Cai, Tianyou Yang, Mingxia Zhang

**Affiliations:** ^1^School of Life Science, Henan Institute of Science and Technology, Xinxiang, China; ^2^Cofco Great Wall Sanggan Winery (Huailai) Co., Ltd., Huailai, China; ^3^Yunnan Engineering Research Center of Fruit Wine, QuJing Normal University, Qujing, China

**Keywords:** Sauvignon blanc wine, non-*Saccharomyces* yeasts, *Saccharomyces cerevisiae*, aroma, *Torulaspora delbrueckii*, *Hanseniaspora uvarum*

## Abstract

Non-*Saccharomyces* yeasts have great potential in improving wine quality, showing personality characteristics, and highlighting the terroir of wine. In this study, we evaluated the impact of simultaneous inoculation with the non-*Saccharomyces* yeasts *Torulaspora delbrueckii* or (and) *Hanseniaspora uvarum* in combination with *Saccharomyces cerevisiae* (EC1118 or VL3) on the aromatic compounds and sensory quality of Sauvignon blanc wines. The growth of yeast groups in the alcoholic fermentation process was tracked using fluorescence *in situ* hybridization. The presence of non*-Saccharomyces* yeast notably impacted the distribution of *S. cerevisiae* and was related to the species of yeast. The co-fermentation of *H. uvarum* and *S. cerevisiae* improved the content of total esters, especially acetate esters. Simultaneous inoculation of *T. delbrueckii* or (and) *H. uvarum* significantly increased the content of total terpenes, especially linalool. Similar results were found for some higher alcohols and organic acids. Sensory evaluation showed that the wines mixed fermentation with *H. uvarum* had significantly tropical fruit aroma characteristics. Citrus and mineral notes, typical aroma characteristics of Sauvignon blanc wine, were enhanced by mixed fermentation strategies with *T. delbrueckii* or (and) *H. uvarum* and different *S. cerevisiae.* Hence, co-fermentation by *T. delbrueckii* or *H. uvarum* combined with *S. cerevisiae* could significantly improve the sensory quality of Sauvignon blanc wine.

## 1 Introduction

The winemaking process is essentially the result of microorganisms (yeasts, bacteria, and fungi) acting on grape must, among which yeasts are considered to have the greatest influence on wine quality (Brysch-Herzberg and Seidel, 2015; de Celis et al., 2022). On account of the diversity of yeast from grapes and the winemaking environment, grape must fermentation is performed predominately by the interaction of yeast population, grape must, and environmental conditions (Brysch-Herzberg and Seidel, 2015; Polizzotto et al., 2016). In the spontaneous fermentation process of wine, the early stage is caused by the action of various non-*Saccharomyces* yeasts with a low fermentative ability, while the later stage of alcoholic fermentation is dominated by *S. cerevisiae* (Urso et al., 2008). Commercial *S. cerevisiae* is commonly added to wine alcoholic fermentations due to its ability to induce robust, rapid, and consistent fermentation, but this has also led to a high degree of assimilation of wine qualities. However, in recent years, an increasing number of non-*Saccharomyces* yeasts, including *Hanseniaspora* spp.*, T. delbrueckii, Schizosaccharomyces pombe*, and *Lachancea thermotolerans*, have received more attention in mixed fermentation with *S. cerevisiae* because these non-*Saccharomyces* yeasts can improve sensory quality of wine through increasing the production of desirable aromatic compounds (Renault et al., 2015; Gao et al., 2022; Wang et al., 2024).

In fact, non-*Saccharomyces* yeasts were traditionally listed as undesirable strains in previous studies due to their weak alcohol resistance and low fermentation ability (Ciani et al., 2010). However, a large number of recent studies have found that non-*Saccharomyces* yeasts produce more noticeable positive effects during winemaking than previously thought (Swiegers and Pretorius, 2005). Furthermore, wine produced by spontaneous fermentation is superior to *S. cerevisiae* single fermentation in terms of complexity, body, aroma, and terroir characteristics (Tofalo et al., 2014). Aroma is one of the important sensory indexes of wine. The aroma of white wine mainly consists of varieties and fermentation aromatic compounds (Puertas et al., 2018). The characteristic aromas displayed by different varieties of grapes are related with the grape-derived aromatic compounds. They are usually present in combined form (glycosyl-terpenes, cysteine-thiols) in grapes. Their precursors can be released during fermentation by the action of yeast (Swiegers and Pretorius, 2005). Compared with *S. cerevisiae*, non-*Saccharomyces* yeasts produced stronger hydrolytic enzyme activities, in which pectin enzymes increase grape juice extraction rate and promote wine clarification, β-glycosidases hydrolyze non-volatile glycoside aroma precursors, proteases increase clarification, esterases facilitate esters to produce important fermented aroma, and lipases decompose grape or autolyzed fat of yeast (Renault et al., 2015; Hu et al., 2018; Puertas et al., 2018; Slaghenaufi et al., 2020). Co-fermentations of *S. cerevisiae* with selected non-*Saccharomyces* yeasts in a controlled manner have proven to produce distinct aroma profiles and improve the complexity of the wine. More particularly, non-*Saccharomyces* yeasts are used as a total or partial alternative to sulphites (Simonin et al., 2018). Microbiological spoilage is a major concern throughout the wine industry, and control tools are limited. The research found that a new killer toxin produced by *T. delbrueckii* with potential biocontrol activity of *Brettanomyces bruxellensis*, *Pichia guilliermondii*, *Pichia manshurica*, and *Pichia membranifaciens* wine spoilage showed glucanase and chitinase enzymatic activities and was suggested to be used as a biocontrol tool in winemaking (Villalba et al., 2016; Canonico et al., 2023). So, spontaneous fermentation using indigenous yeasts, or mixed fermentation of selected non-*Saccharomyces* yeasts with commercial *S. cerevisiae*, has become popular during winemaking (Comitini et al., 2011). The initial studies aimed to inoculate one kind of non-*Saccharomyces* yeast and *S. cerevisiae* mixed fermentation wine, and gradually developed to try more than two kinds of non-*Saccharomyces* species mixed fermentation, and more than one non-*Saccharomyces* inoculation was recommended a potential strategy to improve the aroma diversity and quality of industrial wines (Zhang et al., 2022).

Sauvignon blanc is a refreshing white wine that is beloved for its crisp acidity, zesty tastes, and passion fruit and grapefruit aromas. Commercial *S. cerevisiae* strains Lalvin EC1118 and Laffort VL3 are commonly added for Sauvignon blanc fermentation due to excellent alcohol tolerance and good performance in the winemaking process. Besides commercial strains, *H. uvarum* and *T. delbrueckii* have been recognized as strains that have a positive effect on the sensory characteristics of wine (Renault et al., 2015; Hu et al., 2018; Liu et al., 2018; Muñoz-Redondo et al., 2021; Wang et al., 2024). Simultaneous mixed fermentation of *H. uvarum* or (and) *T. delbrueckii* with *S. cerevisiae* or sequential inoculation can reduce the content of acetic acid and acetaldehyde in wine and improve the yield of esters (Hu et al., 2018; Wang et al., 2024). Hence, the co-fermentation of *H. uvarum* and *T. delbrueckii* with *S. cerevisiae* was performed in this study, with which one or two kinds of non-*Saccharomyces* species were mixed to explore the possibility of improving the sensory quality of Sauvignon blanc wines. There are cell–cell contacts within mixed-culture fermentation. Previous studies have demonstrated that cell–cell contact is crucial for the early death of non-*Saccharomyces* yeasts in the mixed fermentation with *S. cerevisiae*, and the contact between yeast cells could have effects on metabolites (sugar, nitrogen metabolism, ester production) and gene expression levels (Renault et al., 2015; Wang et al., 2024; Zhang et al., 2024). In order to detect the effect of non-*Saccharomyces* yeast inoculated on commercial *S. cerevisiae*, fluorescence *in situ* hybridization (FISH) was applied to track the dynamic change of *S. cerevisiae.* FISH is a direct visualization with the reliability of molecular methods. It was applied to the analysis of yeast population dynamics during alcoholic fermentation (Xufre et al., 2006). The D1/D2 domains of 26S rRNA of yeasts are the ideal basis for yeast-specific FISH probes design, considering that 26S rRNA shows high degree of variability between species. The accessibility map of the D1/D2 regions of the 26S rRNA in *S. cerevisiae* was produced, facilitating the design of non-*Saccharomyces* specific FISH probes (Xufre et al., 2006). Thus, sequences of FAM-labeled oligonucleotide probes targeting *S. cerevisiae* species 26S rRNA D1/D2 region in this study.

## 2 Materials and methods

### 2.1 Grapes and yeast strains

Sauvignon blanc grape was picked up from *Domaine Franco-Chinois* (Huailai, Hebei Province, China) on 17 September 2018. The physicochemical indexes of grapes juice were as follows: total sugar 220 g/L, total acid 7.1 g/L, and pH 3.3.

Four strains, including two *S. cerevisiae* commercial strains EC1118 (Lallemand, France) and VL3 (Laffort, France), and two non-*Saccharomyces* strains (*T. delbrueckii* and *H. uvarum*, obtained from China General Microbiological Culture Collection Center, CGMCC), were used in the present study, and the preservation numbers are 2.4064 (*T. delbrueckii*, TD4064) and 2.4487 (*H. uvarum*, HU4487), respectively. The strains were stored at −80°C in yeast extract peptone dextrose (YPD) medium, with the addition of glycerol (20% v/v final concentration). The YPD medium contains 1% yeast extract (Oxoid, United Kingdom), 2% peptone (Oxoid, UK), and 2% dextrose (Fisher, United States). Before inoculation, sufficient strains were obtained by using a YPD medium to gradually amplify culture. The strain was activated on an agar slant tube of YPD and then gradually expanded and cultured in liquid test tubes and liquid triangular flasks until the strain concentration exceeded 1.0 × 10^9^ CFU/mL. The strain was collected by centrifugation and washed three times with sterile saline. Then the expanded *S. cerevisiae* and non-*Saccharomyces* yeasts were inoculated according to the inoculation scheme. The inoculation amount was 1.0 × 10^6^ CFU/mL for *S. cerevisiae* and 1.0 × 10^7^ CFU/mL for non-*Saccharomyces*, and different strains were simultaneously inoculated at the ratio of 1:10 (*S. cerevisiae* to non-*Saccharomyces* yeasts).

### 2.2 Wine fermentations and physiological characteristics of finished wines

Sauvignon blanc grapes were harvested at optimum ripeness, destemmed, and crushed. Maceration was carried out at 8–10°C for 24 h with the addition of 1 g pectinase (Laffort, France) and 1 mg/L of H_2_SO_3_ (calculated as 60 mg/L SO_2_), and the 8 L of Sauvignon blanc grape juice was transferred to a 10 L fermentor to produce the wine. The strains stored at −80°C were first activated on the YPD agar medium to estimate their viabilities. Then a monocolony was selected and gradually amplified in the YPD medium under 28°C and 150 rpm until the strain concentration exceeded 1.0 × 10^9^ CFU/mL. For Sauvignon blanc fermentation, the inoculation amount was as mentioned above, and different strains were simultaneously inoculated at a ratio of 1:10 (*Saccharomyces* to non-*Saccharomyces*). The fermentation temperature was maintained at 14–16°C. Daily sugar and temperature were monitored to trace the kinetics of the alcoholic fermentation. The alcoholic fermentation would finish when the sugar reduction was less than 10 g/L for two consecutive analyses. Considering that the production of CO_2_ was decreasing and the wine sample was risking oxidation, the wine samples were racked and post-fermented. H_2_SO_3_ was added to the raw wine to the concentration of SO_2_ reached 60 mg/L. The post-fermentation was carried out at 10°C–12°C for 30 days. The supernatant was filtered, bottled, and stored for 3 months. Then, the wine samples were used for sensory analysis and tested related characteristics of reducing sugar, total acid, alcohol, dry extract, and glycerol content according to GB/T 15038-2006 (General analysis method for wine and fruit wine).

During alcoholic fermentation, the single fermentation of two *S. cerevisiae* strains and the mixed fermentation of two non-*Saccharomyces* strains with EC1118 and VL3 strains were carried out as follows: (1) single inoculation with EC1118 (EC1118); (2) simultaneous inoculation of EC1118 and TD4064 (EC1118/TD); (3) simultaneous inoculation of EC1118 and HU4487(EC1118/HU); (4) simultaneous inoculation of EC1118, TD4064 and HU4487(EC1118/TD/HU); (5) single inoculation with VL3(VL3); (6) simultaneous inoculation of VL3 and TD4064(VL3/TD); (7) simultaneous inoculation of VL3 and HU4487(VL3/HU); and (8) simultaneous inoculation of VL3, TD4064, and HU4487(VL3/TD/HU). All fermentations were carried out in duplicate in a 10 L fermentor.

### 2.3 Fluorescence *in situ* hybridization analysis

Fluorescence *in situ* hybridization (FISH) was used for the analysis of the growth and decline of *S. cerevisiae* and non-*Saccharomyces* yeast by detecting and quantifying yeast species in this study. The fluorescent probes were designed in the following sequence: Sce (5′-TGACTTACG TCGCAGTCC-3′, specific to *S. cerevisiae*, labeled with FAM, i.e., fluorescein phosphoramidite); Huv (5′-TCAATCCCGGCTAACAGTA-3′, specific to *H. uvarum*, labeled with quasar670, i.e., quasar 670 phosphoramidite); Tde (5′-GCAGTATTTCTACAGGAT-3′, specific to *T. delbrueckii*, labeled with quasar570, i.e., quasar-570-CE phosphoramidite). *Saccharomyces cerevisiae* and non-*Saccharomyces* yeasts were cultured in the YPD medium and hybridized with respective FISH probes. The strains were cultured for 12 h and were sampled and diluted to OD_600_ = 0.3. The samples were centrifuged for 5 min at 6000 rpm, and then the cells were washed once with phosphate-buffered saline and fixed with 4% paraformaldehyde solution of equal volume for 3 h at 4°C. After the fixed solution was centrifuged at 10,000 rpm for 3 min, 1 mL of hybridization buffer was added to the pellet, which was then centrifuged at 10,000 rpm for 3 min. The pellet was added with 80 μL of hybridization buffer, 80 μL of formamide, and 20 μL of probe, and incubated at 46°C for 3 h. After incubation, cells were precipitated by centrifugation at 10,000 rpm for 3 min, and then the cells were resuspended with 200 μL of phosphate-buffered saline. The rinsed samples were placed under a ZFM-400 fluorescence microscope (Zhengxi Instrument, China) to observe the specific fluorescence signal of the samples, and the growth and decline of yeasts of *S. cerevisiae* during fermentation were analyzed by CytoFLEX flow cytometry (BECKMAN COULTER Life Sciences, America). During Sauvignon blanc wines fermentation, the wines were sampled every 48 h, hybridized with FISH probes and analyzed with flow cytometry as the method mentioned above.

### 2.4 Aromatic compounds analysis

The analysis of aromatic compounds in the final wines was performed by Headspace Solid-Phase Microextraction coupled with Gas Chromatography–Mass Spectrometry (HS-SPME/GC–MS) according to the method described by Zhang et al. (2011). In brief, aromatic compounds were extracted with a 50/30 μm divinylbenzene/carboxen/polydimethylsiloxane fiber (Supelco, Bellefonte, PA, United States). A 5.0 mL of wine, 10.0 μL of 1-methyl-2-pentanol solution (2.0 mg/L, internal standard), and 1.0 g NaCl were held in a 15.0 mL vial that contained a magnetic stirrer, equilibrated at 40°C with stirring for 30 min, extracted for 30 min, and then desorbed in the GC injector at 250°C for 15 min. Each wine sample was carried out in triplicate. Separation of aromatic compounds was carried out on GC–MS (Agilent 6,890 GC-5975B MS, Agilent, United States) with HP-INNOWAX column (60.0 m × 0.25 mm × 0.25 μm, Agilent, United States). GC program: initial temperature 50°C for 1 min, then increased to 220°C at a rate of 3°C/min, and maintained at 220°C for 5 min. Helium was the carrier gas with a purity of 99.999% at a flow rate of 1 mL/min. MS program: mass range 30–500 (m/z), ion source temperature 230°C, and ionization voltage 70 eV. MS was operated in the full scan and the selective ion mode (SIM) under autotune conditions at the same time. Aromatic compounds were identified by comparing their mass spectrum and retention time with those of pure standards using the NIST14 standard reference library. Quantitative analysis was achieved by interpolating the relative areas vs. the area of the internal standard using calibration graphs established for pure standards.

### 2.5 Sensory analysis

The sensory evaluation was conducted in accordance with the method reported by Lan et al. (2021). The trained sensory panel was comprised of 10 assessors (5 females and 5 males) from the College of Enology, Northwest A&F University, China, who had all completed the College’s wine tasting course. The panel sniffed samples to identify aromatic descriptors. After brief training of sensory panelists with reference flavors, each wine sample was evaluated. The sensory panel evaluated the appearance (depth/color intensity, color), aroma, taste (richness/intensity, body, acidity, aftertaste length), and overall rating of each sample. In order to complete the online sensory evaluation of wine samples, the Easy Sensory Analysis System (v2.0), a software developed by the China National Institute of Standardization (CNIS), was utilized. In the online system, sensory panelists were asked to describe the aroma of each wine sample and write a minimum of three and a maximum of five descriptors. The aroma attribute description was described in eight aspects, namely vegetables (green grass, pine leaves, eucalyptus, green pepper), flowers (locust, lily, osmanthus, honeysuckle), citrus fruits (lemon, orange, grapefruit), drupaceous fruits (apple, peach, apricot), tropical fruits (pineapple, mango, litchi), fermentation aroma (bread, cookie, yogurt, dough), aging aroma (mineral, nut, honey), and off-flavor (pickle, wet cardboard, nail polish, dust), respectively. All tasters were instructed to independently complete all tasting texts and were prohibited from communicating with one another during the experiments.

### 2.6 Statistical analyses

One-way ANOVA using the Duncan test at a significance level of *p* ≤ 0.05 was carried out to uncover statistical differences between the wines produced from the different inoculation methods. Principal component analysis (PCA) was carried out using the concentrations of aromatic compounds (OAV ≥ 0.1) to visualize the differences between wines fermented by different fermentation strategies. Statistical analyses were performed with the IBM SPSS 19.0 Statistical Package. Advanced Heatmap barplot was performed using the OmicStudio tools at https://www.omicstudio.cn/tool.

## 3 Results

### 3.1 Growth and decline of yeasts in the co-fermentation process of non-*Saccharomyces* yeast and *Saccharomyces cerevisiae*

**Figure 1:**
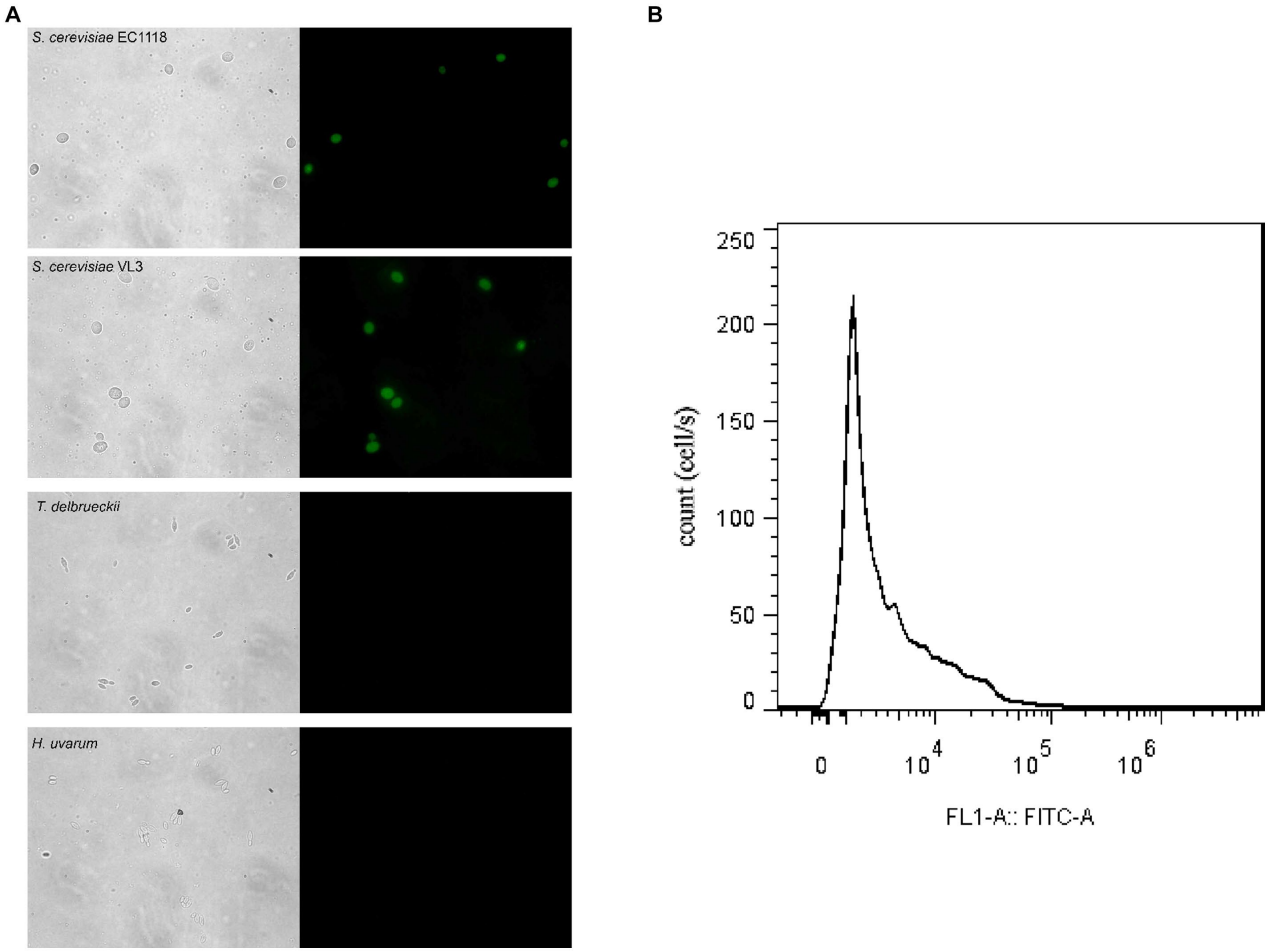
Microscope visualization of fluorescence signals emitted by *Saccharomyces cerevisiae* and non-*Saccharomyces* yeast cells of mixed fermentation samples hybridized with FAM-labeled FISH probes. **(A)** a, b, c, d and a´, b´, c´, d´ correspond to the fluorescence signals of EC1118, VL3, *Torulaspora delbrueckii* and *Hanseniaspora uvarum*, respectively. **(B)** Flow cytometry measurement of *Saccharomyces* in wine samples.

*Saccharomyces cerevisiae* and non-*Saccharomyces* yeasts were cultured in the YPD medium and hybridized with respective FISH probes to detect the specificity of the probes. The results are shown in Figure 1A. The fluorescent probe was specific for EC1118 and VL3 and non-specific for two non-*Saccharomyces* yeast species (Figure 1A), and we could label *S. cerevisiae* by *in situ* hybridization of wine sample fixative and probe. The fluorescence signal could be detected by flow cytometry, and then the *S. cerevisiae* cells with fluorescence signal could be analyzed and sorted (Figure 1B).

The cell sorting was conducted with the same volume and optical density (OD) for the wine sample by flow cytometry. The analysis and sorting of samples automatically ended when the number of cells sorting with fluorescence signal reached 10,000. The cell sorting speed of each wine sample could be calculated by the time it took for the number of cells to reach 10,000 and thus roughly determine the distribution of *S. cerevisiae* in the wine sample. Hence, if the cell sorting speed of the wine sample was faster, it was indicated that the distribution of *S. cerevisiae* in the wine sample was higher. Otherwise, it is the opposite. The cell sorting speed of each wine sample is shown in Table 1.

**Table 1:** Cell sorting speed of each wine sample during flow cytometry analysis (cells/s).

Fermentation strategy	Fermentation time			
	1 day	3 days	5 days	7 days
EC1118	159	484	552	719
EC1118/TD	129	357	443	579
EC1118/HU	127	325	435	545
EC1118/TD/HU	109	278	295	354
VL3	157	455	576	677
VL3/TD	117	324	413	576
VL3/HU	119	304	376	543
VL3/TD/HU	86	167	214	319

With the prolongation of fermentation time, the cell sorting speed of wine samples with different fermentation strategies increased, varying in different degrees (Table 1). Compared with EC1118 or VL3 fermentation individually, the cell sorting speed of wine samples under mixed fermentation conditions was lower than that of the control at the same fermentation time point, indicating that non-*Saccharomyces* yeast inhibited the growth and reproduction of *S. cerevisiae* during alcoholic fermentation. As can be seen from Table 1, when *T. delbrueckii* and *H. uvarum* were separately mixed with EC1118 for alcoholic fermentation, the cell sorting speed showed differences in EC1118/TD and EC1118/HU wines. Moreover, the difference in the degree of competition showed the same result for VL3/TD and VL3/HU wines, which indicated that different types of non-*Saccharomyces* yeasts had different competitive effects on the growth and reproduction of *S. cerevisiae*. In addition, it was also found that in mixed fermentation, whether it was EC1118 or VL3, the inhibition effect of *S. cerevisiae* by two kinds of non-*Saccharomyces* yeasts (TD/HU) was more significant than that by one kind of non-*Saccharomyces* yeast (TD or HU). The reason might be that the competition for nutrients between different kinds of yeast leads to the inhibition of the growth of *S. cerevisiae* in the initial stage. The sorting speed of different wine samples by flow cytometry analysis showed that the presence of non-*Saccharomyces* yeast had a certain influence on the distribution of *S. cerevisiae* during alcoholic fermentation, and the degree of influence generally depended on species of non-*Saccharomyces* yeast, inoculation mode, inoculation amount, and fermentation conditions (Roullier-Gall et al., 2020).

### 3.2 Effect of non-*Saccharomyces* yeast and *Saccharomyces cerevisiae* co-fermentation on the physicochemical characteristics of Sauvignon blanc wine

**Figure 2:**
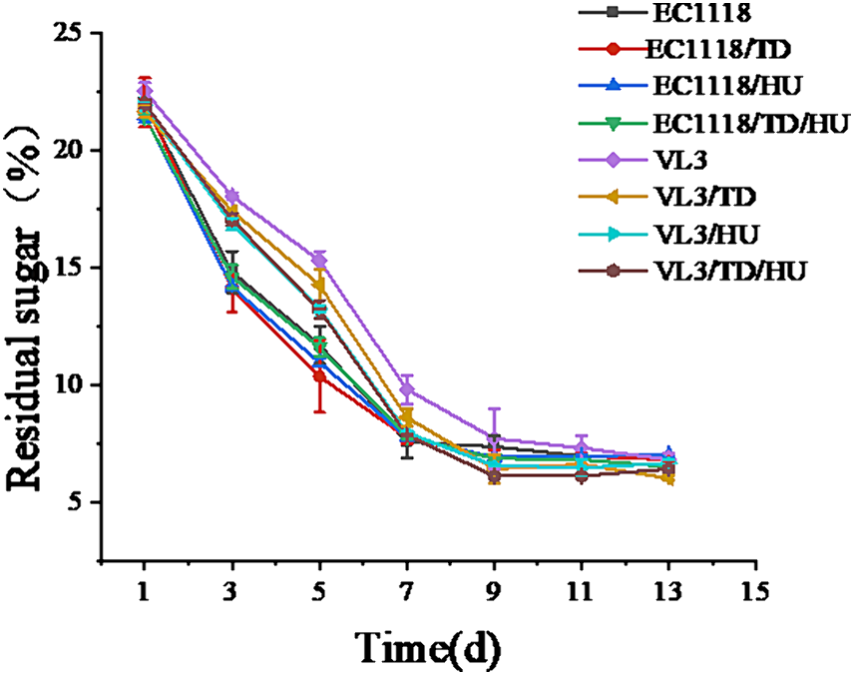
Total sugar change during alcoholic fermentation of Sauvignon blanc wines.

The progress of sugar content was monitored during different fermentation strategies (Figure 2). All alcoholic fermentations were essentially sluggish before the 13th day. Before the seventh day, wines inoculated with VL3 showed a slower sugar consumption rate than those with EC1118. However, sugar consumption in wines inoculated with VL3 combined with non-*Saccharomyces* was faster than in those inoculated with single VL3. Co-fermentation of VL3 with non-*Saccharomyces* accelerated the sugar consumption. Interestingly, the alcoholic fermentations inoculated non-*Saccharomyces* yeasts had relatively higher sugar consumption kinetics than that of single inoculation control at the early stages. However, the sugar consumption began to be significantly lower than control after the ninth day of fermentation. After the 13th day, the sugar reduction was lower than 10 g/L.

The main physicochemical characteristics, including glycerol, dry extract, ethanol, total acids, and residual sugars, are presented in Table 2. Compared to *S. cerevisiae* EC1118 wines, the fermentation of EC1118/TD, EC1118/HU, and EC1118/TD/HU significantly reduced the concentration of glycerol, with the lowest concentration found in EC1118/TD (8.19 g/L). The glycerol content of mixed fermented with *S. cerevisiae* VL3 similarly decreased, ranging from 8.09 g/L (VL3) to 7.64 g/L (VL3/HU). Previous studies have reported that sequential inoculation of *S. cerevisiae* and partial non-*Saccharomyces* yeast mostly increased glycerol content, but the present results showed that simultaneous inoculation of *S. cerevisiae* and two non-*Saccharomyces* yeasts (*T. delbrueckii* and *H. uvarum*) decreased glycerol content in final wines, which was consistent with a previous report (Liu et al., 2018). In agreement with previous studies, lower ethanol contents were observed in the wines simultaneously fermented with *T. delbrueckii* and *H. uvarum* (Renault et al., 2015), but the data showed no statistically significant differences in ethanol concentration among all wine samples.

**Table 2:** Physicochemical characteristics of Sauvignon blanc wines with different fermentation strategies.

Fermentation strategy	Reducing sugar (g/L)	Total acid (g/L)	Alcohol (%, v/v)	Dry extract (g/L)	Glycerol (g/L)
EC1118	5.67 ± 0.01a	6.42 ± 0.10ab	12.88 ± 0.12a	10.80 ± 0.50a	8.67 ± 0.11c
EC1118/TD	5.46 ± 0.01a	7.21 ± 0.31b	12.62 ± 0.38a	14.50 ± 0.30c	8.19 ± 0.10b
EC1118/HU	5.45 ± 0.01a	6.42 ± 0.21ab	12.18 ± 0.58a	12.40 ± 0.80b	8.29 ± 0.15b
EC1118/TD/HU	4.46 ± 0.0b	6.70 ± 0.03ab	12.82 ± 0.16a	12.34 ± 0.25b	8.33 ± 0.13b
VL3	5.95 ± 0.21a	6.65 ± 0.40ab	13.14 ± 0.52a	16.05 ± 0.35d	8.09 ± 0.19b
VL3/TD	5.46 ± 0.05a	6.87 ± 0.13ab	13.04 ± 0.03a	14.80 ± 0.90c	7.69 ± 0.08a
VL3/HU	5.64 ± 0.11a	6.74 ± 0.14ab	12.96 ± 0.05a	14.45 ± 0.55c	7.64 ± 0.10a
VL3/TD/HU	4.72 ± 0.06b	5.94 ± 0.40a	12.74 ± 0.30a	14.45 ± 0.25c	7.70 ± 0.17a

Values are given as mean ± standard deviation of two biological replicates and three detection runs. Data displaying different letters (a, b, c) within each row were significantly different according to the Duncan test at 95% confidence level, while data with the same letter were not significantly different.

In addition, more sugar was consumed by mixed fermentation and the final residual sugar concentrations were further decreased to 4.46 g/L, with the lowest amount in EC1118/TD/HU wine and 4.72 g/L in VL3/TD/HU wine, respectively. Furthermore, no significant differences were observed in the amounts of total acids in different wines.

### 3.3 Aromatic compounds in Sauvignon blanc wine with different fermentation strategies

The aim of this study was to evaluate the diversification of aromatic compounds in Sauvignon blanc wines fermented by non-*Saccharomyces* yeasts (*H. uvarum* and *T. delbrueckii*) and their pairwise combinations with *S. cerevisiae*. Major differences in the concentration of aromatic compounds were found among those final wines, and the detailed results are presented in Table 3. A total of 39 aromatic compounds were quantified, including 15 esters, 15 alcohols, 6 acids, and 3 terpenes, in which the compounds with odor activity values (OAVs) ≥ 0.1 were underlined. Thresholds of compounds were obtained from wine or ethanol solution (Supplementary Table S1).

**Table 3:** Aromatic compound profiles of Sauvignon blanc white wines with different fermentation strategies (μg/L).

Compounds	EC1118	EC1118/TD	EC1118/HU	EC1118/TD/HU	VL3	VL3/TD	VL3/HU	VL3/TD/HU
**Acetate esters**								
Ethyl acetate	10431.8 ± 744.4abc	12551.5 ± 1396.2 cd	16166.1 ± 1150.2e	9935.6 ± 688.6ab	8458.2 ± 155.7a	12329.8 ± 1271.4bcd	17011.5 ± 1033.1e	13642.4 ± 1183.1d
Isoamyl acetate	1165.9 ± 24.6a	1678.3 ± 32.5 cd	1427.7 ± 174.8b	1488.6 ± 79.7bc	1360.7 ± 72.1ab	1714.4 ± 93.3 cd	1503.0 ± 108.8bc	1843.1 ± 97.3d
Hexyl acetate	327.0 ± 20.6 ab	706.0 ± 38.3e	416.7 ± 27.5 cd	391.6 ± 2.9 cd	319.0 ± 18.0a	371.7 ± 15.1bc	383.0 ± 13.7 cd	424.7 ± 10.3d
2-Phenethyl acetate	19.6 ± 1.8a	26.7 ± 0.8 cd	28.0 ± 0.4de	24.7 ± 0.9bc	18.6 ± 1.7a	22.5 ± 0.6b	23.6 ± 1.3b	29.8 ± 0.3e
Total of acetate esters	11944.3 ± 791.4a	14962.5 ± 1467.8b	18038.5 ± 1352.9 cd	11840.5 ± 772.1a	10156.5 ± 247.5a	14438.4 ± 1380.4b	18921.1 ± 1156.9d	15940.0 ± 1291.0bc
**Ethyl esters**								
Ethyl lactate	3122.6 ± 7.5ab	3164.4 ± 30.9b	3101.7 ± 8.5a	3120.6 ± 12.0ab	3101.5 ± 8.9a	3114.8 ± 37.4ab	3136.4 ± 6.7ab	3133.5 ± 31.0ab
Ethyl hexanoate	26.5 ± 1.8e	23.0 ± 0.2d	10.6 ± 0.4b	19.1 ± 1.2c	18.8 ± 1.3c	7.2 ± 1.2a	10.2 ± 0.3b	15.6 ± 2.4c
Ethyl octanoate	60.9 ± 2.0c	50.8 ± 0.2a	54.4 ± 1.4ab	51.4 ± 5.1a	57. 8 ± 3.9abc	59.6 ± 1.7bc	59.2 ± 4.2bc	62.1 ± 2.1c
Ethyl decanoate	10.3 ± 0.4a	13.4 ± 1.4b	20.8 ± 1.8d	14.5 ± 1.1bc	21.2 ± 0.4d	16.8 ± 1.3c	16.7 ± 1.1c	20.5 ± 1.1d
Ethyl dodecanoate	31.9 ± 1.3c	18.2 ± 1.5a	18.6 ± 0.6a	24.3 ± 1.8b	34.1 ± 1.8c	23.7 ± 2.3b	19.4 ± 1.1a	17.8 ± 0.4a
Ethyl tetradecanoate	1.3 ± 0.1d	1.5 ± 0.1d	2.3 ± 0.1e	0.3 ± 0.0a	0.6 ± 0.0bc	0.4 ± 0.0a	0.4 ± 0.0a	0.7 ± 0.1c
Total of ethyl esters	3253.5 ± 13.1ab	3271.3 ± 34.3b	3208.4 ± 12.8a	3230.2 ± 21.2ab	3234.0 ± 16.3ab	3222.5 ± 43.9ab	3242.3 ± 13.4ab	3250.2 ± 37.1ab
**Other esters**								
Diethyl succinate	0.1 ± 0.0a	0.1 ± 0.0a	0.1 ± 0.0a	0.1 ± 0.0a	0.1 ± 0.0a	0.2 ± 0.0ab	0.2 ± 0.0b	0.2 ± 0.0b
Isoamyl hexanoate	2.2 ± 0.1bc	2.1 ± 0.1ab	1.8 ± 0.2a	2.5 ± 0.1c	1.9 ± 0.0a	1.7 ± 0.1a	2.0 ± 0.2ab	1.9 ± 0.1a
Methyl octanoate	0.4 ± 0.0	0.5 ± 0.1ab	4.4 ± 0.2	1.2 ± 0.1	0.4 ± 0.1	0.5 ± 0.0	1.5 ± 0.1	0.4 ± 0.0
Methyl decanoate	52.6 ± 4.5b	38.3 ± 2.5a	73.9 ± 9.5 cd	50.1 ± 3.9ab	74.2 ± 3.7 cd	70.1 ± 4.7c	67.8 ± 6.6c	83.2 ± 3.7d
Methyl salicylate	2.2 ± 0.0bc	2.6 ± 0.1c	2.0 ± 0.2ab	1.9 ± 0.2ab	1.5 ± 0.1a	1.9 ± 0.1ab	2.4 ± 0.1c	3.2 ± 0.3d
Total of other esters	57.5 ± 4.6b	43.6 ± 2.8a	82.2 ± 10.1 cd	55.8 ± 4.3b	78.1 ± 3.9bc	74.4 ± 4.9c	73.9 ± 7.0c	88.9 ± 4.1d
Total of esters	15255.3 ± 809.1a	18277.4 ± 1504.9b	21329.1 ± 1375.8 cd	15126.5 ± 797.6a	13468.6 ± 267.7a	17735.3 ± 1429.2b	22237.3 + 1177.3d	19279.1 ± 1332.2bc
**Higher alcohols**								
2-Methyl-1-propanol	171.2 ± 15.1c	288.9 ± 19.3d	123.2 ± 9.7ab	60.2 ± 5.9a	38.0 ± 1.8a	103.4 ± 9.6b	100.0 ± 4.0b	141.1 ± 6.3b
Butanol	5.2 ± 0.4bc	5.8 ± 0.1c	15.5 ± 0.5d	4.8 ± 0.4abc	4.9 ± 0.5bc	4.5 ± 0.3ab	4.3 ± 0.4ab	3.7 ± 0.2a
2, 3-Butanediol	64.4 ± 4.5c	70.1 ± 0.7c	66.6 ± 5.8c	64.8 ± 1.1c	31.0 ± 4.0a	39.2 ± 3.3a	37.5 ± 2.1a	52.9 ± 4.3b
Isoamyl alcohol	5718.4 ± 43.4b	6608.4 ± 253.1c	6146.4 ± 365.6bc	5820.2 ± 278.0b	4741.2 ± 360.5a	5538.8 ± 409.3b	6209.23 ± 239.8bc	4330.0 ± 238.7a
3-Methylthio propanol	0.7 ± 0.1a	0.9 ± 0.0a	1.4 ± 0.2b	0.6 ± 0.1a	0.6 ± 0.2a	0.8 ± 0.1a	0.7 ± 0.1a	0.9 ± 0.2a
Pentanol	7.1 ± 0.7d	11.4 ± 1.8e	6.5 ± 0.2 cd	5.1 ± 0.1abc	3.3 ± 0.3a	4.6 ± 0.5ab	6.7 ± 0.4 cd	5.6 ± 0.5bcd
Hexanol	44.4 ± 0.0b	87.5 ± 4.2d	40.5 ± 2.1b	37.8 ± 1.5b	12.6 ± 1.5a	41.3 ± 1.9b	58.7 ± 5.0c	94.9 ± 7.9d
3-Methyl-1-pentanol	7.6 ± 0.6bc	8.8 ± 0.6c	5.1 ± 0.6a	6.3 ± 0.5ab	8.6 ± 1.2c	8.9 ± 0.7c	8.2 ± 0.1c	8.5 ± 0.2c
4-Methyl-1-pentanol	2.7 ± 0.4abc	5.0 ± 0.3d	2.7 ± 0.1abc	2.6 ± 0.2ab	2.2 ± 0.0a	3.2 ± 0.3bc	2.9 ± 0.1bc	3.3 ± 0.3c
E-3-Hexenol	1.7 ± 0.1a	2.5 ± 0.2b	1.5 ± 0.1a	1.6 ± 0.1a	1.5 ± 0.0a	1.6 ± 0.1a	1.7 ± 0.1a	2.5 ± 0.2b
3-Ethoxy-1-propanol	6.7 ± 0.6ab	7.3 ± 0.1ab	5.8 ± 0.4a	8.0 ± 0.6b	nd	nd	nd	nd
Octanol	4.5 ± 0.4a	6.7 ± 0.4b	6.9 ± 0.6b	6.6 ± 0.5b	5.0 ± 0.4a	4.9 ± 0.4a	7.1 ± 0.5b	7.4 ± 0.4b
Benzyl alcohol	9.8 ± 1.9ab	10.1 ± 1.4abc	13.2 ± 1.5c	6.9 ± 0.7a	7.2 ± 0.2a	7.5 ± 1.7a	8.5 ± 0.4ab	11.0 ± 1.6bc
2-Phenylethanol	1610.5 ± 82.8abc	1868.1 ± 189.0c	2267.3 ± 204.5d	1385.6 ± 130.9a	1457.4 ± 96.7a	1518.3 ± 109.1ab	1473.2 ± 112.6a	1843.5 ± 152.2bc
Dodecanol	Nd	nd	nd	nd	0.7 ± 0.1b	0.7 ± 0.0b	0.6 ± 0.1b	0.4 ± 0.0a
Total of higher alcohols	7654.9 ± 151.0b	8981.5 ± 471.2c	8702.6 ± 591.9c	7411.1 ± 420.6b	6314.2 ± 467.4a	7277.7 ± 537.3b	7919.3 ± 365.7b	6505.7 ± 413.0a
**Terpenes**								
Linalool	2.6 ± 0.2a	3.5 ± 0.1bcd	3.8 ± 0.1 cd	3.0 ± 0.3ab	2.5 ± 0.2a	3.7 ± 0.2 cd	3.9 ± 0.3d	3.3 ± 0.3bc
Dihydrolinalool	0.5 ± 0.1a	0.6 ± 0.1ab	0.7 ± 0.0abc	0.7 ± 0.1abc	0.5 ± 0.0a	0.6 ± 0.1ab	0.8 ± 0.1bc	0.9 ± 0.1c
Citronella acetate	0.5 ± 0.0a	0.9 ± 0.1c	0.8 ± 0.0a	0.7 ± 0.0a	0.8 ± 0.1c	0.7 ± 0.0bc	0.7 ± 0.1bc	0.9 ± 0.1c
Total of terpenes	3.6 ± 0.3a	5.0 ± 0.3 cd	5.3 ± 0.1d	4.4 ± 0.4bc	3.8 ± 0.3ab	5.0 ± 0.3 cd	5.4 ± 0.5d	5.1 ± 0.3 cd
**Organic acids**								
Acetic acid	296.5 ± 8.1bc	322.3 ± 3.0d	307.9 ± 11.6 cd	282.6 ± 15.8b	233.9 ± 0.2a	240.6 ± 4.5a	242.9 ± 1.2a	251.6 ± 4.5a
2-Methyl propionic acid	213.0 ± 1.1a	214.9 ± 8.1a	213.6 ± 0.1a	211.5 ± 1.4a	nd	nd	nd	nd
3-Methyl butyric acid	212.2 ± 0.4c	212.4 ± 5.9c	213.9 ± 0.1c	210.7 ± 0.3bc	204.3 ± 1.9a	205.8 ± 0.6ab	206.0 ± 0.5ab	208.7 ± 0.2abc
Hexanoic acid	267.7 ± 8.8abc	286.4 ± 9.0 cd	291.7 ± 19.0d	259.9 ± 5.4ab	251.8 ± 0.8a	258.6 ± 12.5ab	263.3 ± 3.0abc	275.9 ± 2.4bcd
Octanoic acid	137.7 ± 1.4a	180.0 ± 4.8c	180.7 ± 10.7c	167.1 ± 1.6bc	128.7 ± 5.4a	161.2 ± 11.2bc	172.8 ± 13.8bc	172.7 ± 1.5bc
Decanoic acid	26.7 ± 1.2a	31.8 ± 2.3b	45.9 ± 2.4e	39.3 ± 2.1 cd	36.9 ± 1.7c	45.7 ± 1.9e	44.0 ± 2.7de	59.4 ± 2.8f
Total of organic acids	1153.8 ± 21.0d	1247.8 ± 33.1e	1253.7 ± 43.9e	1171.1 ± 26.6d	855.6 ± 10.0a	911.9 ± 30.7b	929.0 ± 21.2b	968.3 ± 11.4c

nd, not detected; 13 odor-active compounds (OAV ≥ 0.1) were underlined. Values are given as mean ± standard deviation of two biological replicates and three detection runs. Data displaying different letters (a, b, c, d, e, f, g, h) within each row were significantly different according to the Duncan test at a 95% confidence level, while data with the same letter were not significantly different.

#### 3.3.1 Esters

Esters are the main composition of secondary compounds produced by yeast metabolism during winemaking. The effects of different fermentation strategies on the profile of esters were diverse and complex in Sauvignon blanc wines. Eight of the 15 esters quantified exceeded the individual odor threshold in the study, including 4 acetate esters (ethyl acetate, isoamyl acetate, hexyl acetate, and 2-phenethyl acetate) and 4 ethyl esters (ethyl lactate, ethyl hexanoate, ethyl octanoate, and ethyl decanoate).

The highest concentration of total esters was produced by mixed fermentation with VL3/HU (22237.3 μg/L), and the lowest concentration of total esters was produced by single strain starters with VL3 (13468.6 μg/L) in the VL3 group. A similar phenomenon was found in EC1118/HU fermentation wine, which had the highest concentration of total esters (21329.1 μg/L), and was 1.40-fold higher than EC1118 single fermentation (15255.3 μg/L). It was indicated that the concentration of total ester was significantly increased by *H. uvarum*. Furthermore, different fermentation strategies using different yeast strains had distinct impacts on the formation of specific esters. The combination of *H. uvarum* and EC1118(VL3) further increased the concentration of acetate esters, especially for ethyl acetate, hexyl acetate, and 2-phenethyl acetate, in which the concentrations were increased by 55.0% (101.0%), 27.4% (20.1%), and 42.9% (26.9%), compared to those fermented by the EC1118 (VL3) control, respectively. For another, mixed fermentation using *H. uvarum* and EC1118 (VL3) did not remarkably increase the concentration of ethyl esters compared with the control (*p* ≤ 0.05). As shown in Table 3, only the concentration of ethyl decanoate increased in wine-inoculated EC1118/HU, and the reason might be that *H. uvarum* produced more decanoic acid, which was precursor forming ethyl decanoate (Table 3). However, the mixed fermentation of VL3/TD/HU significantly increased total esters compared with VL3 in Sauvignon blanc wine.

The effect of *T. delbrueckii* with EC1118 (VL3) mixed fermentation on esters was also investigated. Simultaneous inoculation of *T. delbrueckii* and EC1118 (VL3) significantly increased total esters, especially acetate esters, including ethyl acetate, isoamyl acetate, hexyl acetate, and 2-phenethyl acetate. For ethyl esters, there was no significant difference (*p* ≤ 0.05). Furthermore, there was no significant difference in total esters between EC1118/TD/HU and EC1118 fermentation wine.

#### 3.3.2 Higher alcohols

Fifteen higher alcohols were quantified (Table 3) in Sauvignon blanc wine, and two compounds (isoamyl alcohol and 2-phenylethanol) exceeded their odor thresholds. The highest total concentrations of higher alcohols were produced by mixed fermentation with EC1118/TD (8981.5 μg/L). Nevertheless, the lowest was VL3 single fermentation (6314.2 μg/L). The result also showed that EC1118 monoculture could produce higher alcohols than VL3, revealing that different strains of *S. cerevisiae* produce different levels of higher alcohols. Co-inoculation of *T. delbrueckii* with EC1118 significantly increased the total of higher alcohols compared with EC1118 single inoculation and the highest contents (8981.5 μg/L) were found in EC1118/TD wine. Specifically, co-inoculation of EC1118 with *T. delbrueckii* significantly improved the production of higher alcohols (*p* ≤ 0.05), including 2-methyl-1-propanol, isoamyl alcohol, pentanol, hexanol, E-3-hexenol, and 4-methyl-1-pentanol, octanol. The EC1118/TD wine produced the highest concentration of isoamyl alcohol, which was 15.6% higher than that of EC1118 single fermentation (*p* ≤ 0.05), but the content of isoamyl alcohol in VL3/HU fermentation wine increased by 31.0% compared with that of VL3 (*p* ≤ 0.05), which was higher than that of VL3/TD. The results revealed that different strains of *S. cerevisiae* caused the difference in the content of higher alcohols. Meanwhile, the competition between *S. cerevisiae* and different species of non-*Saccharomyces* yeast in the process of winemaking also resulted in a difference in the content of higher alcohols.

#### 3.3.3 Terpenes and volatile organic acids

Terpenes are derived from grapes and have a major positive impact on the floral aroma of wines, either directly or through synergistic effects (Swiegers and Pretorius, 2005; Slaghenaufi et al., 2020). Three terpenes were detected and one compound with OAV > 0.1 (linalool, sweet, and floral note). As shown in Table 3, a significant increase in the terpene level was found after inoculation with non-*Saccharomyces* yeasts (*p* ≤ 0.05). It was found that simultaneous inoculation of *T. delbrueckii* and *H. uvarum* significantly increased the content of total terpenes, especially the content of linalool. Terpenes are generally present in grapes in the form of glycoside conjugates (such as arabinoside, rhamnoside, and glucoside). In the winemaking process, the release of terpenes requires corresponding glycosidases (such as α-L-arabinofuranosidase, α-L-rhamnosidase, or β-D-apiosidase) to hydrolyze monoterpene-β-D-glucoside, and release terpenes through β-D-glucosidase. Many studies have shown that the β-glycosidases activity of non-*Saccharomyces* yeasts is higher than that of *S. cerevisiae* (Padilla et al., 2016). Therefore, co-fermentation with non-*Saccharomyces* yeasts can release more terpene. This study is consistent with the conclusions of previous literature, verifying that both *T. delbrueckii* and *H. uvarum* have higher β-glycosidases activity, which releases more terpene aroma and increases the complexity of wine aroma (Polizzotto et al., 2016; Gao et al., 2022).

Six organic acids were detected in Sauvignon blanc wine, including acetic acid, 2-methyl propionic acid, 3-methyl butyric acid, hexanoic acid, octanoic acid, and decanoic acid. Compared with *S. cerevisiae* single fermentation, mixed fermentation with *H. uvarum* significantly increased the content of organic acids, such as octanoic acid, decanoic acid, and hexanoic acid. Furthermore, compared with EC1118 single fermentation, mixed fermentation with *T. delbrueckii* significantly increased the concentrations of acetic acid, octanoic acid, and decanoic acid. For wines inoculated VL3, mixed fermentation with *T. delbrueckii* significantly increased the concentrations of octanoic acid, decanoic acid, and 2-methyl propionic acid, which were not detected in VL3 fermentation wines.

### 3.4 Multivariate analysis and sensory analysis of Sauvignon blanc wine with different fermentation strategies

**Figure 3:**
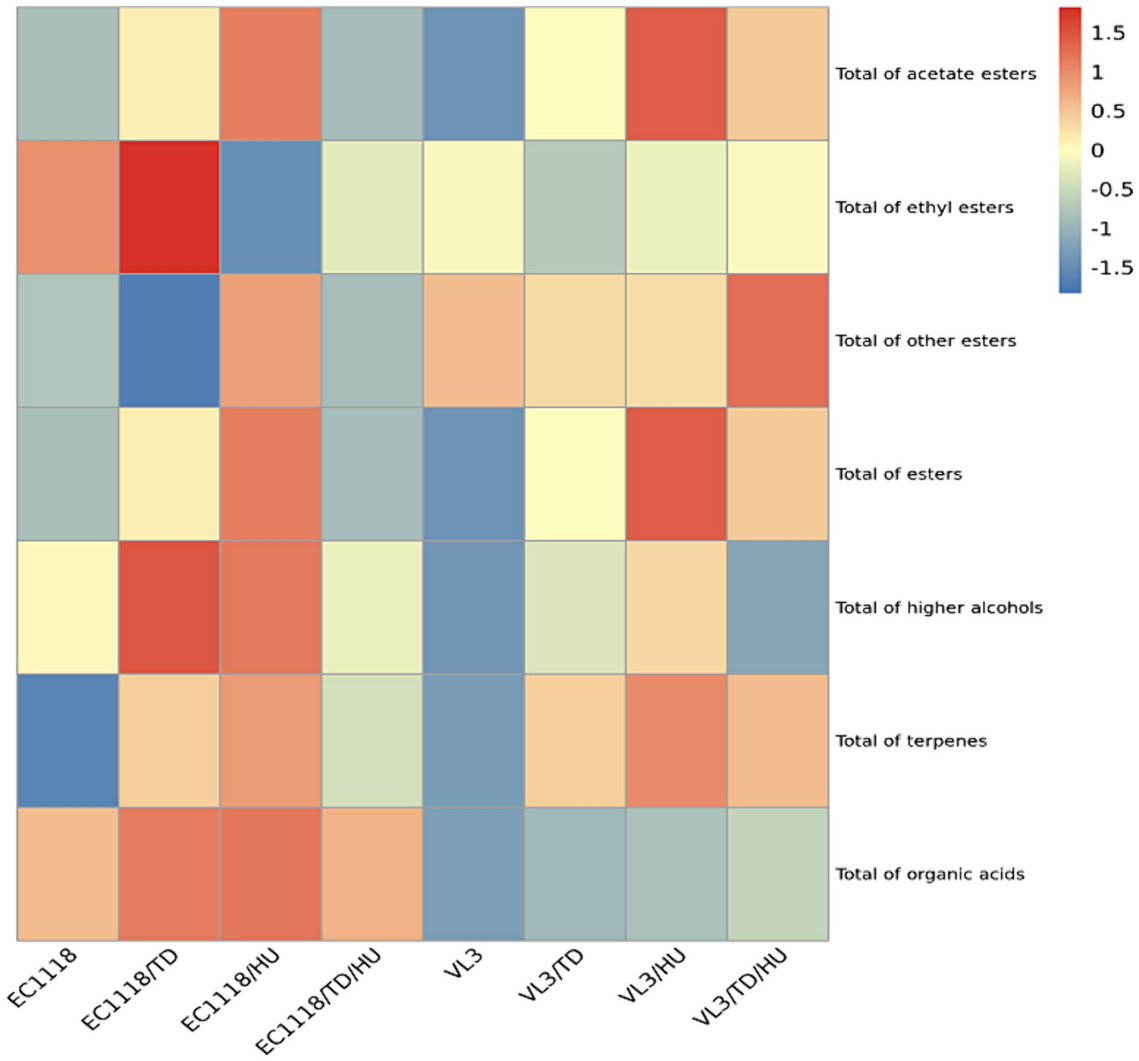
Heat maps of different types of aromatic compounds under different fermentation strategies.

The heat map showed that mixed fermentation of *T. delbrueckii* and *H. uvarum* with *S. cerevisiae* could increase the level of acetate esters (Figure 3), especially *H. uvarum*. The result was consistent with previous reports (Zhang et al., 2023; Valera et al., 2024). For ethyl esters, the influences of *T. delbrueckii* and *H. uvarum* with EC1118 co-fermentation showed no significant difference (Figure 3). This is similar to those ofVL3. Furthermore, non-*Saccharomyces* yeasts, *T. delbrueckii* and *H. uvarum,* can both increase the total ester content in wines fermented by different *S. cerevisiae* stains, and the contribution of *H. uvarum* to total ester was superior to that of *T. delbrueckii*. These results indicated that both *T. delbrueckii* and *H. uvarum* could increase the content of aromatic compounds in Sauvignon blanc wine, which was beneficial to improving the flavor of wine. In terms of higher alcohols, the inoculation of *T. delbrueckii* and *H. uvarum* could increase the total higher alcohol content. Higher alcohols produced by *H. uvarum* and *S. cerevisiae* co-fermentation were higher than those by *T. delbrueckii*. Terpenes can give grapes and wine rich and unique aromas, such as rose, osmanthus, pineapple, and honey, and increase the complexity and coordination of wine (Swiegers and Pretorius, 2005). It was found that the content of terpenes in Sauvignon blanc wine was not high; only linalool exceeded the taste threshold, and *T. delbrueckii* and *H. uvarum* could increase the content of total terpenes.

**Figure 4:**
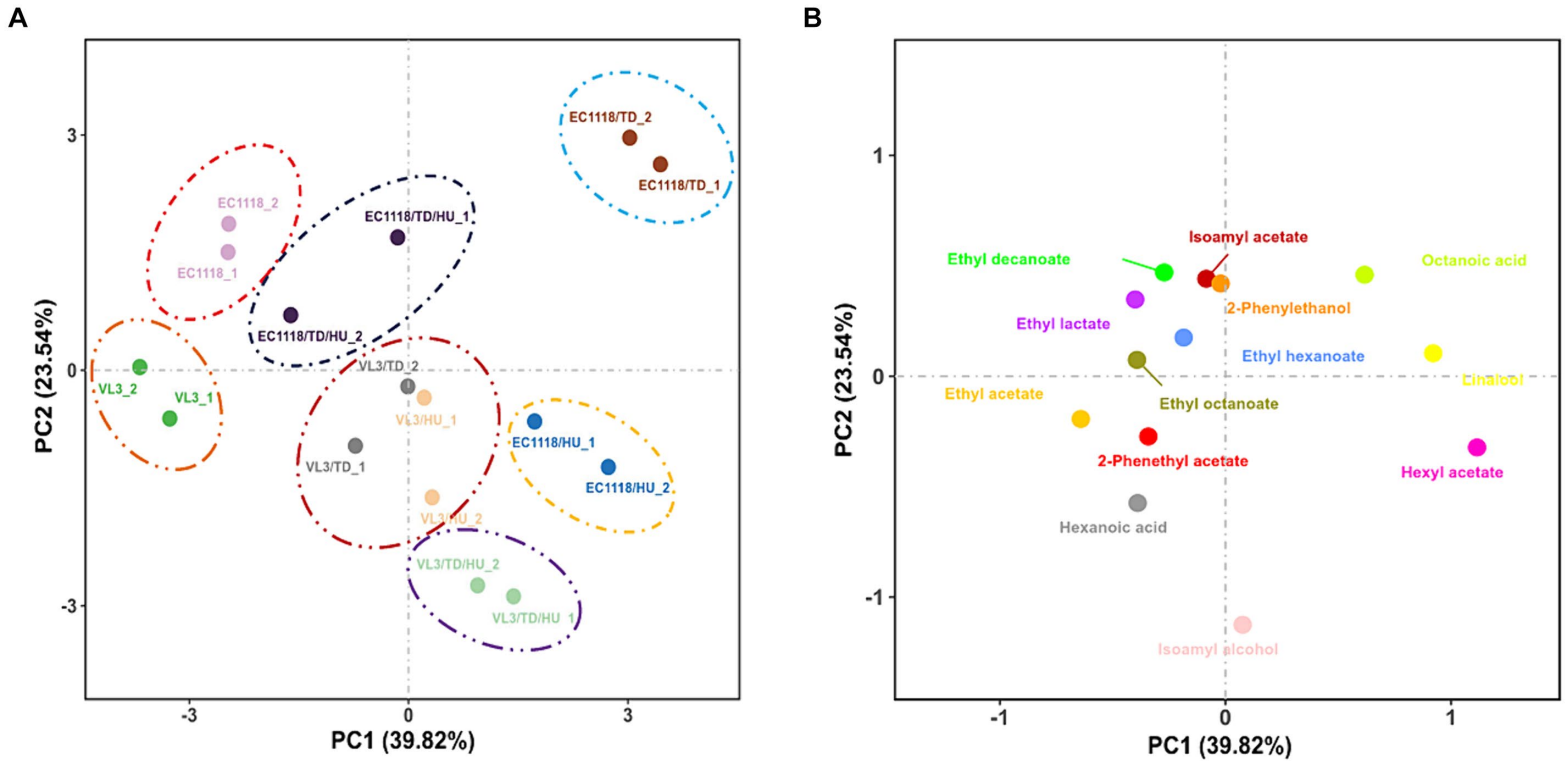
**(A)** Score plot and **(B)** loading plot of principal component analysis of the aromatic compounds (OAV ≥ 0.1) in final Sauvignon blanc wines with eight fermentation strategies.

PCA was carried out to highlight the divergences of fermentation strategies and 13 aromatic compounds (OAV ≥ 0.1) in final Sauvignon blanc wines by eight wine starters (Figures 4A,B). The first two components (PCs) explained 63.36% of the variability, with PC1 accounting for 39.82% and PC2 accounting for 23.54%, respectively. The PCs roughly distinguished wine samples fermented by different inoculation strategies. The Sauvignon blanc wines detected were clustered well, showing high experimental reproducibility.

Based on the data obtained with PCA (Figure 4A), it should be remarked that the inoculation of *T. delbrueckii* indeed brought about significant differences in the aromatic profile of Sauvignon blanc wines across PC1, especially in the case of mixed fermented wines involving EC1118, and the wines mixed fermented with the VL3 strain could also be further separated from each other across PC1. According to Figure 3, mixed fermented wines of E1118/TD were distinctly separated by PC1 and PC2, especially by PC1 from the other wines, suggesting that EC1118/TD had a higher potential to produce a distinct aromatic profile than that of EC1118. The main responsible aromatic components for this separation were linalool, hexyl acetate, octanoic acid, and 2-phenethyl acetate. In terms of VL3 wines, mixed fermented with *T. delbrueckii* and *H. uvarum* were also clearly separated by PC1, which showed that *T. delbrueckii* and *H. u*var*um* were responsible for aromatic components, ethyl acetate, hexyl acetate, 2-phenethyl acetate, and linalool (Figure 4B). The control of EC1118 fermented wines was positioned in the negative direction of PC1 and was associated with lower concentrations of acetate esters, higher alcohols, and terpenes. Wines were fermented by *T. delbrueckii* and *H. uvarum*, and their combinations could be separated by PC1. While the high loadings of many acetate esters and higher alcohols were detected in the positive part of PC1, mainly related to *T. delbrueckii* and *H. uvarum*.

### 3.5 Sensory analysis

**Figure 5:**
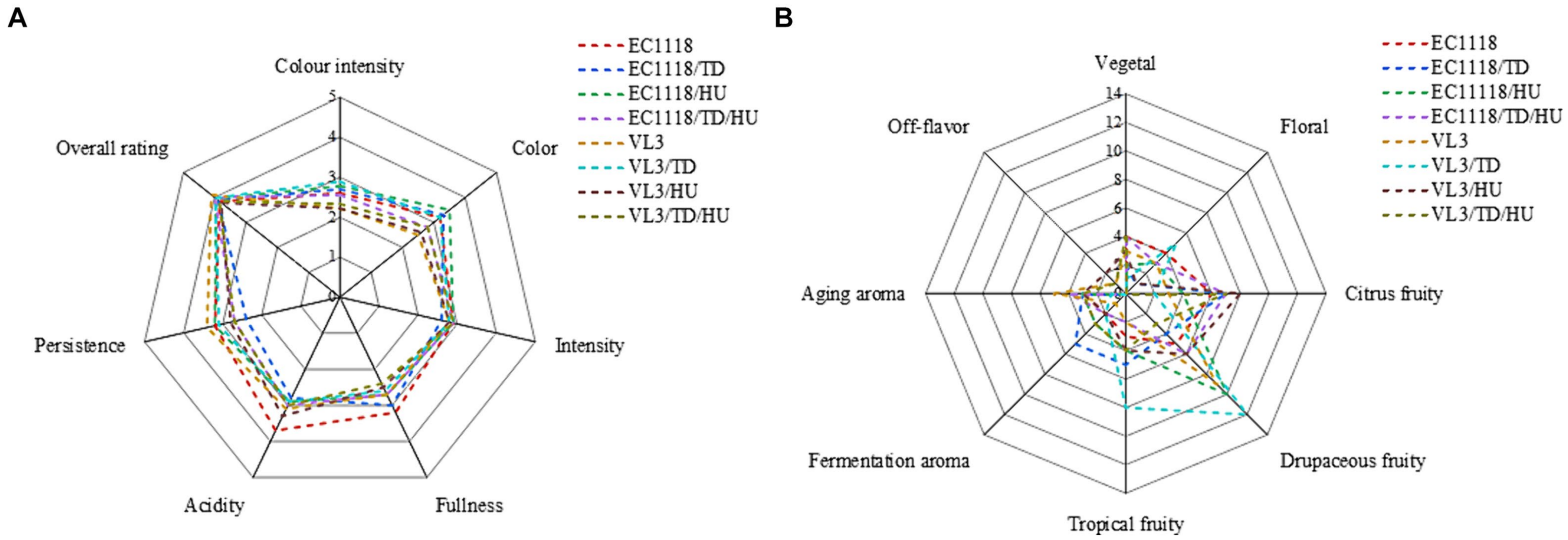
Cobweb diagram of the sensory scores for Sauvignon blanc wines with different fermentation strategies. **(A)** Sensory scores for each wine and **(B)** absolute frequency of descriptors among the terms used by the panel.

Sauvignon blanc wines were evaluated in terms of appearance, fullness, scent intensity, persistence, and fragrance, and the organoleptic analysis showed that the fermentative strategy impacted the sensory perception of the Sauvignon blanc wines (Figure 5). Compared with EC1118 wine, the EC1118/HU wine’s persistence increased, but the fullness slightly decreased and the color became greener. Similarly, the final wine fermented by EC1118/TD showed the same characteristics, and it could be confirmed that the scent intensity of wine fermented by *T. delbrueckii* mixed with *S. cerevisiae* increased, especially in drupaceous fruits (peaches, apricots, apples, etc.). However, compared with the EC1118 monoculture, the mixed fermentation with *H. uvarum* increased the aroma of flowers and fruits significantly, especially osmanthus aroma, white flowers aroma, tropical fruit aroma and drupaceous aroma. Furthermore, the combination of *H. uvarum* and VL3 further increased the aroma of tropical fruit, including isoamyl acetate (banana) and *T. delbrueckii* also enhanced fruit aromas compared to VL3 monoculture, including ethyl acetate, 2-phenethyl acetate, and ethyl lactate, but mainly in the citrus and drupaceous aromas.

Furthermore, the citrus aroma was not affected in EC1118/TD/HU wine, but the aroma of flowers was inhibited to a certain extent. Compared with VL3 monoculture, the aroma of citrus fruits and vegetables in VL3/HU wine significantly increased compared with VL3 wine, while the aroma of flowers and tropical fruits in VL3/TD wine decreased, but the aroma of drupaceous fruit increased. Additionally, fruits and vegetable aroma and aged aroma increased in VL3/TD/HU wine, but the aroma of flowers reduced. In conclusion, when non-*Saccharomyces* yeasts and *S. cerevisiae* were mixed in fermentation, there might be synergistic or masking effect between aroma due to different strains used.

## 4 Discussion

Wine fermentation is a complex process that involves many physiological and biochemical reactions and the interaction of various yeast growth and metabolism activities (Tofalo et al., 2014; Tzamourani et al., 2023). At present, there are limited monitoring methods for the spatial and temporal distribution of microorganisms during wine fermentation. Some scholars have monitored the types and quantities of microorganisms in different fermentation stages through the spreading plate method (Lleixà et al., 2016; Zhang et al., 2024), and some have used molecular biological methods such as restriction fragment enzyme digestion and microsatellite PCR (Zhang et al., 2023). In addition, metagenomics has also been used to analyze the abundance of microorganisms during winemaking (de Celis et al., 2022; Goppold et al., 2023), but metagenomics has an advantage over identifying bacterial species and is more difficult to identify species and genera of yeast. Therefore, the selection of appropriate microbial species and quantity monitoring methods is of great significance for studying the influence of microorganisms on the wine fermentation process and their contribution to flavor compounds.

The FISH technique was used to study the interaction of yeast in the process of winemaking (Xufre et al., 2006; Branco et al., 2012). Because the fluorescence signal of specific yeast strains can be marked by *in situ* hybridization reaction, the distribution of microorganisms in wine samples can be analyzed quickly and accurately, and the interaction between strains in the process of winemaking can be judged. Flow cytometry was used to analyze the cell sorting rate of different wine samples to determine the distribution of *S. cerevisiae* in wine samples. The cell sorting rate of different wine samples showed a great difference due to the different fermentation strategies. As the inoculation amount of non-*Saccharomyces* increases, it further hinder the growth of *S. cerevisiae*. No matter whether EC1118 and VL3 were inoculated with *T. delbrueckii* or *H. uvarum*, the amount of *Saccharomyces* in the wine sample was obviously smaller than that in a single *S. cerevisiae* inoculation. The distribution of *S. cerevisiae* was affected by the presence of non-*Saccharomyces* yeasts during winemaking. Unfortunately, due to the poor specificity of non-*Saccharomyces* yeast probes designed, the growth and decline of *T. delbrueckii* and *H. uvarum* could not be traced well. The result was in agreement with previous reports (Villalba et al., 2016; Roullier-Gall et al., 2020). It was supposed that non-*Saccharomyces* yeasts could produce toxic compounds, such as glycoproteins, peptides, and small molecular weight signal molecules, which inhibit the growth of *S. cerevisiae* at the initial fermentation stage (Villalba et al., 2016; Zhang et al., 2024).

In the Sauvignon blanc wine alcoholic fermentation process, the co-inoculation of non-*Saccharomyces* yeasts (*T. delbrueckii*, *H. uvarum*) and *S. cerevisiae* affected the speed of sugar consumption before the ninth fermentation day. Simultaneous inoculation of *T. delbrueckii* or (and) *H. uvarum* with *S. cerevisiae* accelerated alcoholic fermentation, and this phenomenon was more obvious in VL3 mixed fermentation. The reason for the difference may be that non-*Saccharomyces* yeasts have different effects on the growth and reproduction of different types of *S. cerevisiae* strains (Lleixà et al., 2016). Moreover, it is well known that Lalvin EC1118 has strong adaptability and fermentation ability. The flow cytometry analysis proved that when inoculated with non-*Saccharomyces*, the growth of EC1118 was less inhibited compared with that of VL3. It is not sensitive to the winemaking environment (Deed et al., 2017). It was also noted that the glycerol content decreased in wines with mixed fermentation. Similar results were reported (Liu et al., 2018; Zhang et al., 2021). Although some authors reported that sequential inoculation of non-*Saccharomyces* yeast with *S. cerevisiae* increased the glycerol of wine (Muñoz-Redondo et al., 2021). The results showed that the glycerol profiles of the final wines were changed when simultaneously inoculated with non-*Saccharomyces* yeast and *S. cerevisiae*, indicating that the inoculation method might affect the glycerol content in the final wines during the mixed fermentation of *S. cerevisiae* and non-*Saccharomyces* yeast. However, transcriptomic analysis of differences between the two species for the genes involved in the glycerol pathway showed that *T. delbrueckii* lacked GPD2 and GPP2 (Tondini et al., 2019).

In winemaking, one or more non-*Saccharomyces* yeasts are artificially inoculated, in order to play a role in highlighting wine characteristics and improving wine quality. Non-*Saccharomyces* yeasts influence the aroma and sensorial properties of white wine. Among the aromatic compounds in wines, esters play a vital role in shaping the aroma and taste profile of wines. In this study, EC1118 (VL3) with *T. delbrueckii* or (and) *H. uvarum* co-inoculation increased the levels of total ester and acetate esters in Sauvignon blanc wines, enhancing the fruits and flowers characteristics of the wines, especially for VL3 with two non-*Saccharomyces* yeasts. *Hanseniaspora uvarum* was considered a high acetate ester producer (Liu et al., 2020; Zhang et al., 2023). In this study, *H. uvarum* combined with EC1118 (VL3) produced a wine with higher levels of acetate esters, including ethyl acetate, isoamyl acetate, hexyl acetate, and 2-phenethyl acetate, when compared to that of EC1118 and VL3 single inoculation, respectively. These findings were in agreement with several previous studies that also reported significant increases in ester content (Liu et al., 2020; Zhang et al., 2023; Wang et al., 2024; Zhang et al., 2024), such as ethyl acetate, isoamyl acetate, and 2-phenethyl acetate. Renault et al. (2015) proposed that the enhancement of esters content in the mixed *T. delbrueckii* with *S. cerevisiae* wine fermentation was due to positive interactions between *S. cerevisiae* and non-*Saccharomyces* yeast (Renault et al., 2015).

Higher alcohols contribute to the complexity of the wine aroma due to their concentration under 300 mg/L (Swiegers and Pretorius, 2005). Different strains of *S. cerevisiae* caused significant differences in the content of higher alcohols. Compared to EC1118, VL3 and *T. delbrueckii* or (and) *H. uvarum* co-fermentation improved the level of higher alcohols in wines. *Torulaspora delbrueckii*, whether mixed with EC1118 or VL3 co-inoculation, could almost increase the amount of all alcohols, except for butanol and octanol in VL3. The reason may be attributed to positive interactions between *S. cerevisiae* and *T. delbrueckii* (Renault et al., 2015). Higher alcohols are produced mainly in the process of yeast alcohol fermentation (Swiegers and Pretorius, 2005). The two species of yeasts promote each other in favor of the accumulation of alcohol. Isoamyl alcohol (burnt, malt, and whisky notes) and 2-phenylethyl alcohol (with floral and rose notes) were the main higher alcohols based on their concentrations. The co-fermentation of *T. delbrueckii* or *H. uvarum* with *S. cerevisiae* improved the level of two alcohols, while simultaneous inoculation of *T. delbrueckii* and *H. uvarum* with *S. cerevisiae* could not increase the amount of both alcohols at the same time. The results are consistent with a previous report on the combined use of *S. cerevisiae* and more than one non-*Saccharomyces* species (Zhang et al., 2022).

Many studies have shown that non-*Saccharomyces* yeasts secrete glycosidase, hydrolyzing glycoside-terpenes from grapes, and furthermore, increasing the number of free terpenes in wine (Swiegers and Pretorius, 2005; Slaghenaufi et al., 2020; Gao et al., 2022). In this study, a similar result was found that simultaneous inoculation of *T. delbrueckii* or (and) *H. uvarum* significantly increased the content of total terpenes, especially the content of linalool. Acids play an important role in wine fermentation, not only directly determining the flavor features of the produced wines but also influencing the biosynthesis of ethyl esters (Zhang et al., 2022). Two non-*Saccharomyces* yeasts inoculated also affected the content of organic acids in wines. Compared with *S. cerevisiae* single fermentation, mixed fermentation with *T. delbrueckii* or (and) *H. uvarum* increased the content of organic acids, such as octanoic acid, decanoic acid, and hexanoic acid. As a result, the content of total organic acids increased in wine.

Sensory analysis showed the difference between *T. delbrueckii* or (and) *H. uvarum* with *S. cerevisiae* fermented Sauvignon blanc wine and that of single *S. cerevisiae*. OAVs are commonly used to evaluate the contribution of volatile compounds to the aroma of wine (Benkwitz et al., 2012; Deed et al., 2017). The difference was associated with aromatic compounds with OVA ≥ 0.1 in this study. Esters mainly contribute characteristics of flowers and fruits to wine aroma. *Hanseniaspora uvarum* was considered a high acetate ester producer (Swiegers and Pretorius, 2005; Liu et al., 2020; Zhang et al., 2023). In this study, *H. uvarum* combined with EC1118 (VL3) produced a wine with higher levels of acetate esters, including ethyl acetate, isoamyl acetate, hexyl acetate, and 2-phenethyl acetate, compared to that of EC1118 and VL3 single inoculation, respectively. Former studies have revealed tha Sauvignon blanc wines are characerized by high hexyl acetate (apple, cherry, and pear) and isoamyl acetate (banaan, fruity) (Benkwitz et al., 2012). In our study, the OAV of isoamyl acetate in wine samples exceeds 200. The results of sensory evaluation showed that the wines mixed fermentation with *H. uvarum* owned significant tropical fruit aroma characteristics. Citrus and mineral notes are considered to be typical aroma characteristics of Sauvignon blanc wine, and thiols are known as significant contributors. They are present in trace amounts in wine, so they could not be detected by conventional equipment in this study (Tan et al., 2021). However, the citrus and mineral notes of Sauvignon blanc were identified during sensory evaluation. VL3 was more able to highlight the aroma characteristics of Sauvignon blanc wines. The mixed fermentation with *T. delbrueckii* or (and) *H. uvarum* enhanced the typical aroma characteristics of Sauvignon blanc wines.

## 5 Conclusion

In this study, we have demonstrated mixed fermentation of two non-*Saccharomyces* yeasts, *H. uvarum* or (and) *T. delbrueckii* with *S. cerevisiae* was a good fermentation strategy to improve the sensory quality of Sauvignon blanc wines. Moreover, simultaneous inoculation allowed for the improvement of the Sauvignon blanc dry wine’s aroma complexity, increasing the esters, and higher alcohol and terpenes content in the wines. Compared to *S. cerevisiae* single culture, simultaneous inoculation of *H. uvarum* (*T. delbrueckii*) efficiently increased the production of most of the desired compounds associated with fruits, flowers, and sweet characteristics, enhancing the aromatic diversity of Sauvignon blanc wines.

## Data availability statement

The original contributions presented in the study are included in the article/Supplementary material, further inquiries can be directed to the corresponding author.

## Author contributions

LL: Conceptualization, Data curation, Writing – original draft, Writing – review & editing. CY: Formal analysis, Writing – review & editing. LZ: Formal analysis, Writing – review & editing. RC: Investigation, Writing – review & editing. QY: Resources, Writing – review & editing. JC: Formal analysis, Writing – review & editing. TY: Writing – review & editing. MZ: Conceptualization, Funding acquisition, Writing – original draft, Writing – review & editing.
