# Supplementary material for: The impact of simultaneous inoculation with Torulaspora delbrueckii and Hanseniaspora uvarum combined with Saccharomyces cerevisiae on chemical and sensory quality of Sauvignon blanc wines
Source: Front Microbiol. 2024 Jul 24;15:1413650. doi: 10.3389/fmicb.2024.1413650 (PMC11303216; doi:10.3389/fmicb.2024.1413650)
Supplement: Supplementary file 1 [file Table_1.DOCX]

Supplementary Material

# SupplementaryTables

Table. S1 Aroma descriptor and threshold

| **Compounds** | **Aroma descriptor** | **Aroma threshold (mg/L)** |
| --- | --- | --- |
| **ACETATE ESTERS** |  |  |
| Ethyl acetate | VA, nail polish, fruity | 7.5 |
| Isoamyl acetate | banana, fruity | 0.05 |
| Hexyl acetate | apple, cherry, floral, pear | 0.4 |
| 2-Phenethyl acetate | honey, rose, tobacco | 0.25 |
| **ETHYL ESTERS** |  |  |
| Ethyl lactate | floral, fruity | 154.0 |
| Ethyl hexanoate | apple peel, fruity | 0.045 |
| Ethyl octanoate | pear, ripe banana, sweet | 0.6 |
| Ethyl decanoate | floral, fruity | 0.2 |
| Ethyl dodecanoate |  |  |
| Ethyl tetradecanoate |  |  |
| **OTHER ESTERS** |  |  |
| Diethyl succinate | fruity | 1250.0 |
| Isoamyl hexanoate |  |  |
| Methyl octanoate |  |  |
| Methyl decanoate |  |  |
| Methyl salicylate |  |  |
| Total of other esters |  |  |
| HIGHER ALCOHOLS |  |  |
| 2-Methyl-1-propanol |  |  |
| Butanol |  |  |
| 2, 3-Butanediol |  |  |
| Isoamyl alcohol | burnt, malt, whisky | 30.0 |
| 3-Methylthio propanol | potato, sweet | 0.5 |
| Pentanol |  |  |
| Hexanol | cut grass, green, floral, resin | 1.1 |
| 3-Methyl-1-pentanol |  |  |
| 4-Methyl-1-pentanol |  |  |
| E-3-Hexenol | cut grass, green | 1.0 |
| 3-Ethoxy-1-propanol |  |  |
| Octanol |  |  |
| Benzyl alcohol | fruity | 200.0 |
| 2-Phenylethanol | honey, lilac, rose, spice | 14.0 |
| Dodecanol |  |  |
| **TERPENES** |  |  |
| Linalool | Rose | 0.025 |
| Dihydrolinalool |  |  |
| Citronella acetate |  |  |
| **ORGANIC ACIDS** |  |  |
| Acetic acid |  |  |
| 2-Methyl propionic acid |  |  |
| 3-Methyl butyric acid |  |  |
| Hexanoic acid | cheese, spicy, sweat | 0.42 |
| Octanoic acid | cheese, goaty, soapy | 0.5 |
| Decanoic acid | fatty, rancid | 1.0 |
